# Advanced Optical Sensing of Phenolic Compounds for Environmental Applications

**DOI:** 10.3390/s21227563

**Published:** 2021-11-14

**Authors:** Ines Delfino, Nadia Diano, Maria Lepore

**Affiliations:** 1 Dipartimento di Scienze Ecologiche e Biologiche, Università della Tuscia, I-01100 Viterbo, Italy; 2 Dipartimento di Medicina Sperimentale, Università della Campania “Luigi Vanvitelli”, I-80138 Napoli, Italy; nadia.diano@unicampania.it (N.D.); maria.lepore@unicampania.it (M.L.)

**Keywords:** phenols, optical methods, biosensors, sensors

## Abstract

Phenolic compounds are particularly dangerous due to their ability to remain in the environment for a long period of time and their toxic effects. They enter in the environment in different ways, such as waste from paper manufacturing, agriculture (pesticides, insecticides, herbicides), pharmaceuticals, the petrochemical industry, and coal processing. Conventional methods for phenolic compounds detection present some disadvantages, such as cumbersome sample preparation, complex and time-consuming procedures, and need of expensive equipment. Therefore, there is a very large interest in developing sensors and new sensing schemes for fast and easy-to-use methods for detecting and monitoring the phenolic compound concentration in the environment, with special attention to water. Good analytical properties, reliability, and adaptability are required for the developed sensors. The present paper aims at revising the most generally used optical methods for designing and fabricating biosensors and sensors for phenolic compounds. Some selected examples of the most interesting applications of these techniques are also proposed.

## 1. Introduction

Over the last several decades, water pollution has threatened both quality of life and public health worldwide. In particular, phenol and related phenolic compounds (chloro, bromo, nitro, and alkyl phenol) that are discharged into the wastewater and can contaminate surface, ground, and sometimes drinking water have recently drawn attention due their potential impact on human and environmental health. They derive from urban, agro-industrial, and livestock-related human activities [1,2], and they can cause adverse effects in all the food chain rings, even at low concentration (μg/L–ng/L). By way of example, Table S1 shows the permissible concentration limit allowed for some phenolic compounds.

The US Environmental Protection Agency (EPA) and European Commission have already placed them on the Priority Pollutants List that must be monitored by local governments in the next years [3,4]. However, it must be considered that the types and mixtures of pollutants can change according to the urban and industrial activities specific for each territory and that synergic effects can strengthen toxic effects. For these reasons, it is crucial to monitor their levels in samples deriving from local wastewaters, to test their biological activity and to develop new sustainable and green strategies to remove them.

Due to their persistence in the environment and their toxicity, the phenolic compounds can induce acute and chronic hazardous health effects [5,6,7,8]. Long-term exposure to phenols can cause irregular breathing, muscle weakness, and respiratory arrest at lethal doses in humans. Chronic exposure to phenols leads to disorders of the gastrointestinal and central nervous systems and the liver, as well as growth retardation and abnormal development and reproduction in animals. It is known that some of phenol compounds affect the endocrine system, altering the hormones balance within the human body [9]. The alteration of correct levels of endogenous hormones or the introduction of chemicals that can mimic their effect in the cell has been related not only to estrogen-dependent tumors but also to an increased risk for a tremendous number of pathologies, such as Alzheimer’s disease, metabolic syndrome, and cardiovascular disorders [10]. The xenoestrogen compound bisphenol A (BPA) is one of the most widely used chemicals, commonly used in the production of epoxy resins, polycarbonates, dental fillers, food storage containers, baby milk containers, and mineral water containers. Due to the extensive manufacturing of these products, human exposure to BPA through several routes, such as food and the environment, is ubiquitous [11,12]. Biomonitoring studies around the world have shown that BPA exposure is common among the general population, with a detectable concentration in more than 80% of the considered cases [13,14,15].

The most largely used techniques for phenolic compounds detection are gas chromatography, high-performance liquid chromatography, and capillary electrophoresis [16]. These well-established methods present some disadvantages, such as cumbersome sample preparation complex and time-consuming procedures. and the need for expensive equipment; consequently. they cannot be used for routine analysis.

For all these reasons, there is a very large interest in developing sensors and new sensing schemes for monitoring the phenolic compound concentration in environments, with special attention to water. Good analytical properties (sensitivity, accuracy, reproducibility, rapidness, and signal-to-noise ratio), reliability (long life, resistance to the environment, and operational safety), and adaptability (small size, light weight, simple structure, and low cost) are required to the developed sensors [17].

In this framework, the optical sensing scheme can play a pivotal role since sensors can be small, light, chemically inert, non-toxic, and immune to electromagnetic interferences. In fact, optical sensors are among the most versatile sensing devices; they can detect a large class of physical and chemical parameters, such as temperature, pressure, force, electric and magnetic field, pH, strain, chemical concentration, displacement, humidity, and many others. 

In 2013, Rodionov et al. [17] revised the development and applications of optical sensors for the determination of phenolic compounds in the period of 1993–2013. They presented an overview of the different approaches for optical sensing with particular attention to spectrophotometric and fluorescent sensors using optical fibers for the delivery and collection of light signals. In their Tables 1 and 2, the authors reported the spectrophotometric and fluorescence sensors for different phenolic compounds with the information about linearity range, limit-of-detection (LOD), and response time for the most representative results reported in the literature in the examined period (see Ref. [17] and cited references). In Table 3 of the cited paper, many optical sensors used in the determination of phenolic compounds in real samples, such as residential and industrial wastewater and river water, were summarized.

The aim of the present paper is to revise the most generally used optical methods for designing and fabricating biosensors and sensors for phenolic compounds with particular attention to the methodologies that we personally used in our previous investigations [18,19,20,21,22]. We also discuss some representative examples for each of them. As far as the cited references, the list is far from being exhaustive, but it is indicative of the large amount of literature available in this field.

## 2. Optical Detection Techniques

A large number of optical techniques can be used for developing fast, accurate, and sensitive sensors for phenol detection. In this section, we briefly summarize some aspects of the most-used ones.

**Figure 1:**
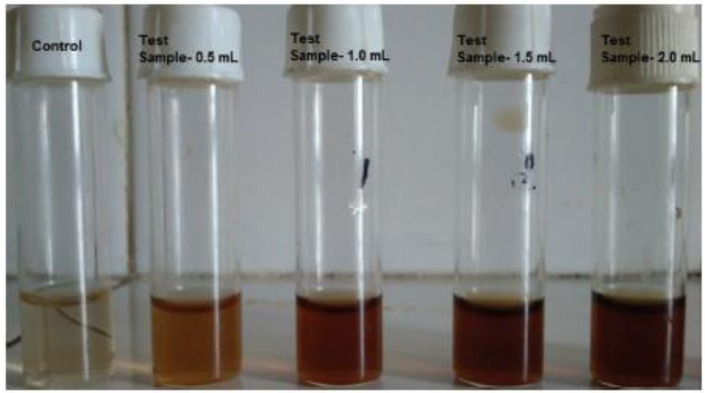
An example of the use of *Streptomyces*-mediated gold nanoparticles for the detection of phenols from industrial wastewater. (Reprinted from [23] under an Open Access condition).

The simplest method is based on the visual inspection of the investigated samples after their interaction with a proper probe. For example, in Figure 1 it is possible to note the color change occurring in water samples with different concentrations of phenols after the interaction with extracellular gold nanoparticles on which *Streptomyces tuirus* DBZ39 has been synthetized (see Ref. [23] for further details).

The qualitatively appreciable color change upon visual inspection can be quantified thanks to absorption measurements. Using a spectrophotometer, it is possible to quantitatively determine absorbance changes linearly related to the presence of different concentrations of phenolic compounds. Bayram et al. [24] show the results obtained by a colorimetric assay based on the formation of quinone-type complexes in an alkaline medium for different concentrations of BPA (see Figure 3 in Ref. [24]). As is evident, different absorbances are obtained in correspondence of color variations, and considering the absorbance values at a particular wavelength, it is possible to obtain a calibration curve that can be used for estimating analyte concentration in other samples of interest. 

**Figure 2:**
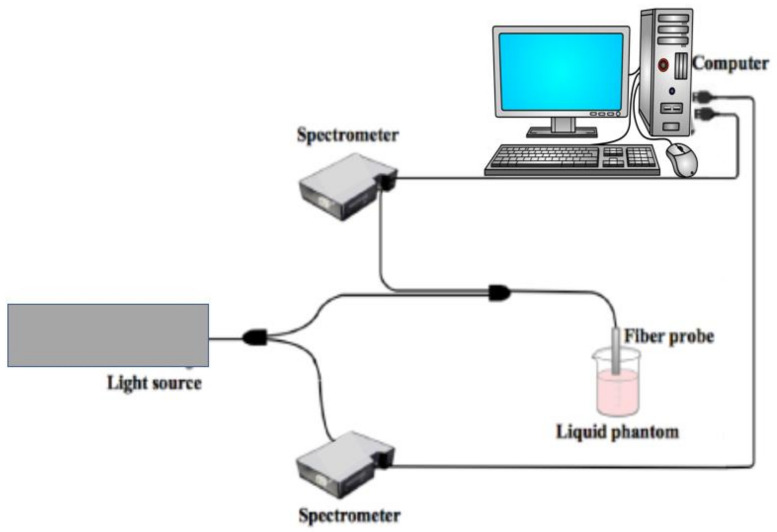
Schematic layout of an experimental set-up for diffuse reflectance measurements. A broadband light source is used, and a spectrometer allows wavelength selection of the incident beam. The second spectrometer is employed for monitoring the reflected signal.

Diffuse reflectance measurements often coupled with the use of optical fibers are also widely used for developing phenolic compounds sensors [25,26]. A typical set-up, where a tungsten halogen broadband is used as a light source and a fiber-optic probe is in contact with the sample used for this approach, is shown in Figure 2. 

**Figure 3:**
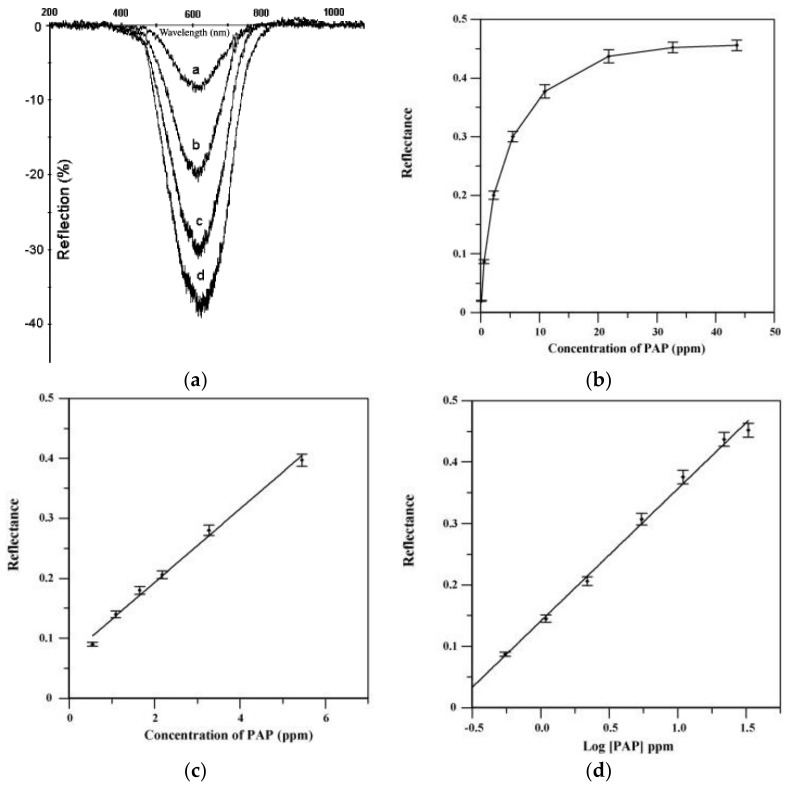
(**a**) Reflectance spectra of immobilized CAL4 before (Rf) and after (a–d) reaction with different concentrations of PAP, being 0.545 mg/L (curve a), 2.18 mg/L (curve b), 5.45 mg/L (curve c), and 10.9 mg/L (curve d). (**b**) Response curve to a wide range of PAP concentrations. The panels (**c**,**d**) show the linear and non-linear portions of the response curve. (Reprinted-adapted-with permission from [25]).

One of the spectrometers is used for recording the lamp spectrum and its background, while the second spectrometer detects the signal reflected the sample. CCD array detectors and a computer allowed the detection and processing of optical signals. In Figure 3a, typical results obtained using this reflectance approach are reported. In these cases, the authors [25] have developed a fiber-optic sensor for p-aminophenol (PAP) based on the use of 25,26,27,28-tetrahydroxycalix[4]arene (CAL4) immobilized onto Amberlite XAD-16 and reflectance spectrometry. The sensor is based on the reaction of PAP with CAL4 in the presence of an oxidant to produce an indophenol dye. The measurements were carried out at 620 nm since it gives the largest differences in reflectance spectra before and after reaction with the analyte. Figure 3b shows the changes in the reflectance spectra of immobilized CAL4 before and after reaction with different concentrations of PAP. In Figure 3c,d, the calibration curves for the wide range of PAP concentrations are reported.

**Figure 4:**
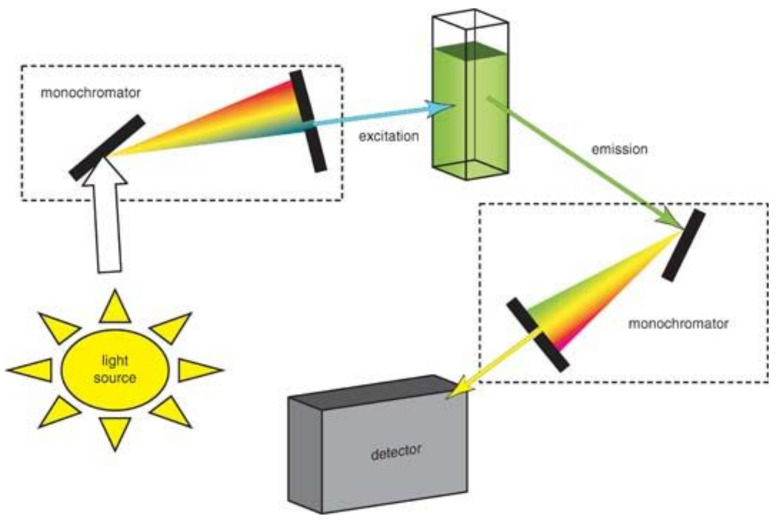
Schematic diagrams of a spectrofluorometer (image taken from https://chem.libretexts.org/Bookshelves/Analytical_Chemistry/Physical_Methods_in_Chemistry_and_Nano_Science_(Barron) (accessed on 8 November 2021).

Fluorescence and luminescence are certainly the most-used methods to develop sensor devices for phenolic substance and to conceive new sensing schemes. See, for example, Table 2 in the review of Rodianov et al. [17] to realize the variety of the available schemes. The fluorescence approach makes available a large cohort of parameters to be used; among them, the most largely used are intensity, decay time, anisotropy, quenching efficiency, and luminescence energy transfer. The components of a basic fluorescence experimental set-up are the light source, two wavelength selectors, and the detector. Usually, the light sources are tungsten-halogen and xenon lamps. As far as the wavelength selection, the simplest apparatus uses fixed filters to isolate both the excitation and emission wavelengths, but it is better to use monochromators to select both the excitation and emission wavelengths. Most modern instruments of this type employ diffraction grating monochromators for this purpose. Using monochromators, both excitation and emission spectra can be recorded making full use of the analytical potential of the fluorescence approach. Regarding detectors, the most largely used are photomultipliers exploiting the wide variety of types available nowadays. Usually, the different components can be chosen and assembled in accordance with the user’s needs, or they are combined in a commercial spectrofluorometer, such as the one reported in Figure 4, in which two monochromators are used as wavelength selectors.

**Figure 5:**
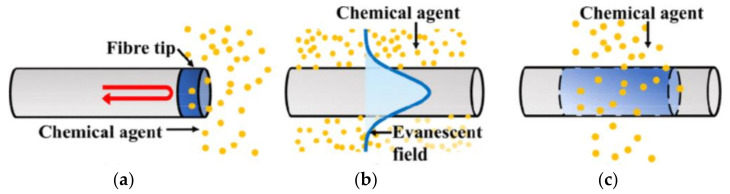
Basic sensing schemes for typical fiber-optic chemical sensors based on (**a**) fiber tip, (**b**) evanescent field sensing, and (**c**) simple transmission setup. (Reprinted from [34] under Open Access conditions).

As is evident from the literature, optical fibers and waveguide-based optical sensors have gained a large interest since they show enormous potential for applications in various fields [27,28,29,30,31]. In fact, fiber-optic sensors can have small size; are sensitive to multiple environmental parameters; allow remote sensing also into normally inaccessible areas; do not strictly require contact; are independent from radio frequency and electromagnetic interference; avoid contamination of their surrounding area; are characterized by high sensitivity, resolution, and dynamic range; and can be connected with data communication systems. A particularly interesting use of optical fibers is related to the development of fiber-optic chemical sensors (FOCS) [32,33]. These devices usually include three main components: an active sensing element that recognizes the analyte and generates an optical signal, a detector that measures one of the characteristics of the optical signal (intensity, frequency, phase) that can be employed for evaluating the concentrations of the analyte of interest, and a computer and a software for data acquisition and processing. In Figure 5, basic sensing schemes for FOCS are shown [34]. In detail, in Figure 5a, one end of the fiber is made sensitive to the chemical substance to be detected. The interaction between the tip of the fiber and the substance causes fluorescence or other signals that can be revealed in the coming back light. In Figure 5b, another configuration for FOCS is reported. In this case, the interaction occurs between the evanescent portion of the light propagating in the fiber and the chemical substances that are near the fiber surface. In Figure 5c, a transmission geometry is adopted for studying the investigated interaction process.

**Figure 6:**
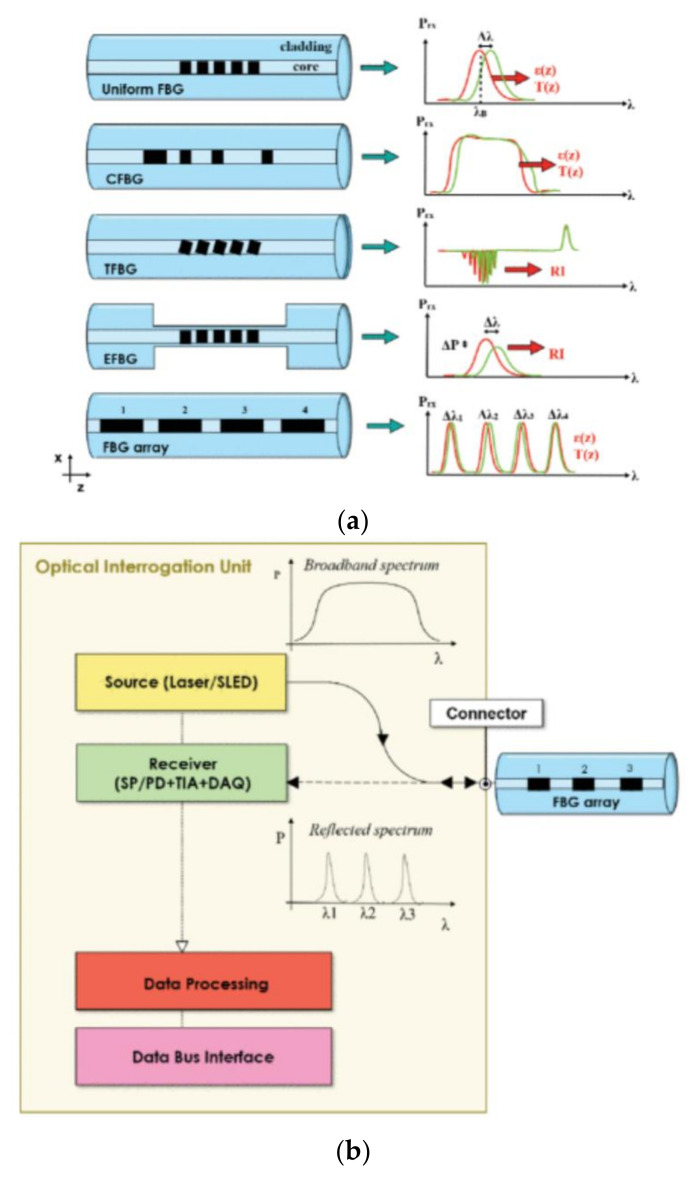
In the (**a**) panel, the main configurations of FBGs are shown. The spectral responses of the measured parameters (strain (ε), temperature (T), refractive index (RI)) are also reported. In the (**b**) panel, a schematic representation of the developed device with all the components used with FBGs is presented. (Reprinted-rearranged-with permission from [36] under Open Access conditions).

Another largely employed approach that uses optical fiber is represented by fiber Bragg gratings (FBGs) that nowadays are used for many applications in different fields by adopting one of the most common configurations, shown in Figure 6 [35,36].

Basically, an FBG-based sensor consists of an optical resonator located inside the fiber-optic core. By using an external laser source and a proper optical element (an interferometer or a diffraction grating), it is possible to periodically perturb the refractive index of a single-mode optical fiber, inducing an interference between waves propagating in the opposite direction in the fiber. The refractive index and the periodicity of the interference pattern can be modified by the external environment, and these changes can be used for mechanical, biomechanical, surgical, physiological, and chemical applications [35,36,37,38].

**Figure 7:**
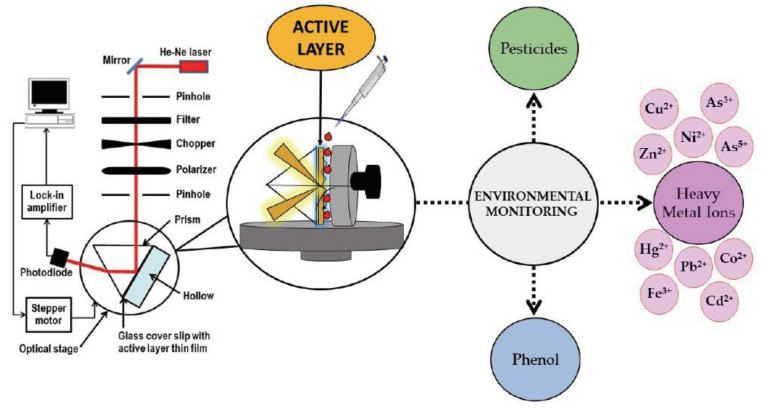
Schematic representation of an SPR optical sensor for phenol and pesticide detection in environmental applications. (Reprinted from [39] under Open Access conditions).

Surface plasmon resonance (SPR) optical sensors offer large advantages in terms of detection limit, sensitivity, and selectivity when compared with other sensing schemes, and for this reason, many researchers have focused their attention on these sensing devices, starting in the 1990s [39]. The SPR effect was observed by Wood in 1902, when he sent a monochromatic polarized light beam on a diffraction grating and he noticed a pattern of white and dark bands. The physical explanation of these effects was reported by Otto and Kretschmann [40]. The SPR configuration proposed by Daniyal et al. [39] is the most-used (see Figure 7) one, and it is based on the “angular interrogation” approach, since the wavelength of the incident light is kept constant, and the angle of incidence of the light is varied. In fact, in this configuration, a monochromatic and p-polarized light is used for exciting a surface plasmon that propagates along a metal surface. At a certain angle, named the resonance angle, the intensity of the reflected light decreases due to resonance, which occurs when the momentum of the surface plasmon wave is equivalent to that of the incident light. This sensing method is based on the incident angle interrogation, since the wavelength of the light is kept constant, and the angle of incidence of the light is varied (angular interrogation) while in the other method, the wavelength of light is varied, and the angle of incidence is kept constant and greater than the critical angle (wavelength interrogation). An SPR optical sensor is based on the measurement of the refractive index near the metal surface. Any changes in refractive index will also change the resonance angle.

To further enhance their sensitivity and selectivity towards a specific target pollutant, various active layers to place on the top of a metal surface have been investigated in the last several years [40].

In the last several decades, there has also been a very large use of vibrational spectroscopies techniques for developing very sensitive optical sensing schemes for phenolic substances. These spectroscopies represent a class of analytical techniques that give information on vibrational energy levels associated with the functionality of the examined sample. Fourier Trasform Infrared (FT-IR) spectroscopy measures the light absorption using a broadband light source in the 4000–500 cm^−1^ wavenumber region (or in the 2500–20,000 nm wavelength range), while Raman spectroscopy (RS) is related to the inelastic scattering process occurring when light interacts with matter [41]. When FT-IR and the Raman spectrometer are equipped with a microscope, they allow the biochemical characterization of samples also at microscopic levels [42,43].

**Figure 8:**
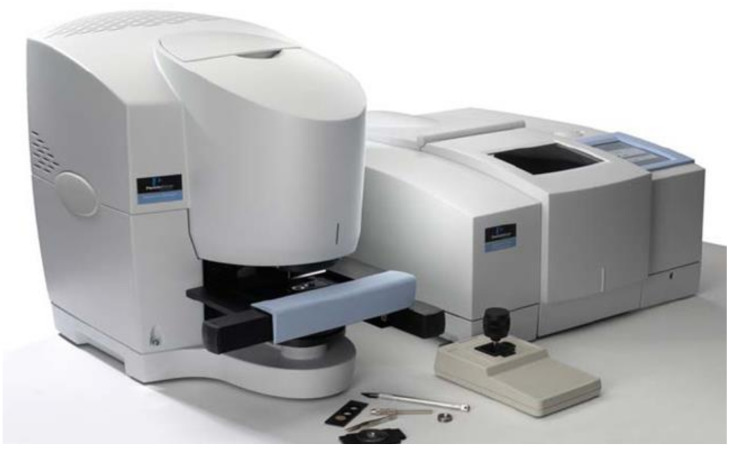
Photo of a commercial Fourier transform infrared (FT-IR) spectrometer equipped with different options for acquisition of spectra in different geometries at micro and macroscopic levels.

In Figure 8, a typical commercial instrument for FT-IR spectroscopy is reported. This system adopts a long-life source with proprietary hot-spot stabilization as a source, and it is equipped with two different detectors for the acquisition at macroscopic and microscopic levels. It allows the acquisition of infrared spectra in transmission and attenuated total reflection (ATR) mode for macroscopic samples. At the microscopic level, spectra can be acquired in transmission, reflectance, transflection, and micro-ATR collection geometry. The availability of all these different approaches makes FT-IR spectroscopy very versatile and suitable for all types of samples [41].

**Figure 9:**
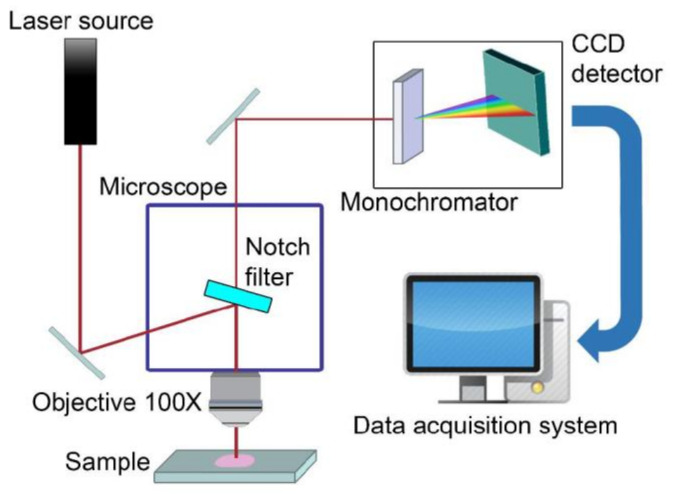
Experimental set-up for micro-Raman spectroscopy.

In Figure 9, a typical micro-Raman set-up is shown. Typical equipment for micro-Raman spectroscopy requires a laser source, a microscope objective to view the sample, focusing the laser beam on the sample and collecting the weak light from it in back-scattering geometry, a filtering system for rejecting the exciting light (Notch filter), a dispersive apparatus (monochromator), a high-sensitivity detector for the detection of the output signal (typically a nitrogen-cooled CCD), an electronic counting chain for the acquisition of the Raman signal, and a software for recording and processing Raman spectra. The adoption of confocal microscope stages allows the acquisition of appreciable Raman signals in experimental conditions of interest for phenolic compound sensing. See Ref. [43] and references therein for additional information.

RS and FT-IR can give complementary information since the related signals are due to nonpolar and polar functional groups, respectively. In addition, the design and fabrication of nanostructured substrates has allowed the development of companion techniques, such as Surface-Enhanced Infrared Radiation Absorption (SEIRA) [22,44,45] and Surface-Enhanced Raman Spectroscopy (SERS) [46], that are currently used for the preparation of a new generation of sensors.

## 3. Biosensors

In discussing optical biosensors, we followed the classification made by Borisov and Wolfbeis in ref. [47] that divide biosensors in two classes: catalytic and affinity biosensors. Catalytic biosensors use biocomponents capable of recognizing biochemical species and causing their transformation in a product by means of chemical reaction. The most relevant examples of this class are enzymatic biosensors. Affinity biosensors use analyte able to bind to a biorecognition element. This class is divided into immunosensors (which exploit the specific interactions between an antibody and an antigen), biosensors based on interactions between an analyte and a bioreceptor, nucleic acid biosensors (which use the affinity between complementary oligonucleotides), and whole-cell biosensors that behave as recognition elements that respond to substances by expressing a specific gene. Some examples of these two different classes of biosensors that have been specifically designed for phenolic species detection in the environment will be reviewed in the following sections.

### 3.1. Enzymatic Biosensors

The enzymes generally used for phenolic compounds biosensing are laccase, tyrosinase, and, in a few cases, horseradish peroxidase. Tyrosinase is characterized by low stability and a relevant inhibition caused by reaction products. Horseradish peroxidase requires hydrogen peroxidase for carrying out its catalytic action. Laccase can catalyze electron-transfer reactions without the presence of cofactors; it is stable and can oxidize phenols and o,m,p-benzenediol compounds when molecular oxygen is present. These characteristics are particularly useful for the development of high-quality biosensors.

#### 3.1.1. Laccase-Based Biosensors

Laccases (benzenediol oxygen oxidoreductase; EC 1.10.3.2) are cuproproteins and are also called polyphenol oxidase or blue multicopper proteins and are widely distributed in higher plants, fungi, and bacteria [48]. Most laccases are characterized by four copper atoms per functional unit, which are crucial for catalytic activity. Copper atoms are allocated in different binding sites and have different spectroscopic, functional, and paramagnetic features that enable their classification into three groups. The copper of type 1 (T1) shows a maximum in the absorption spectrum around 600 nm, and it causes the typical blue color of these cuproproteins. It is the primary site of oxidation. The copper of type 2 (T2) shows only a weak absorption in the visible, and it is electron paramagnetic resonance (EPR) active. The two copper atoms of type 3 (T3) are characterized by an absorption band around 330 nm and are EPR silent [49,50]. In a typical laccase reaction, a phenolic substrate is subjected to one-electron oxidation. The obtained species can be converted to a quinone in the second step of the oxidation process. Low-substrate specificity is typical of laccases, and they show a large variability in their catalytic properties in dependence of the source. Laccase can catalyze the oxidation of many compounds, such as hydroquinone, catechol, guaiacol, 2,6-dimethoxyphenol, polyphenols, aromatic amines, benzenethiols, and a series of other compounds [51].

In 2015, M.M. Rodriguez-Delgado et al. [52] revised the laccase-based biosensors for the detection of phenolic compounds. The authors briefly revised the different immobilization procedures available for the laccase enzyme (see Table 1 of Ref. [52]) and presented also the different optical biosensors reported in the literature.

For phenols detection in environmental analysis, Abdullah et al. [53] developed an optical biosensor exploiting the ability of laccase to oxidize methoxy phenols in the presence of 3-methyl-2 benzothiazolinonehydrazone (MBTH) to produce azo-dye compounds. Stacked films of MBTH in hybrid Nafion/sol-gel silicate and laccase in chitosan were used for fabricating the proposed biosensor that is more selective to catechol, as compared with another analyte, such as guaiacol, o-cresol, and m-cresol, that were also investigated. This characteristic is due to the immobilization of laccase in a hybrid material. The linear range obtained for this biosensor was 0.5–8 mM, and synthetic samples were also used for mimicking real samples.

Sanz et al. [54] developed a laccase-polyacrilamide sensor exploiting absorption and fluorescence and characterized by a linear range of 0.109–2.5 mM and a limit of detection (LOD) of 100 μM. This biosensor was also tested on wastewater samples.

Andreu-Navarro et al. [55] proposed another interesting optical sensing approach for the determination of polyphenols, such as catechol, resorcinol, hydroquinone, pyrogallol, hydroxyhydroquinone, phloroglucinol, and gallic acid. This biosensor is based on the inhibition of the green indocyanine fluorescence in the presence of laccase and positively charged gold nanoparticles caused by polyphenols. The developed chemical system is based on the ability of these substances to delay the oxidation of green indocyanine in the presence of laccase. When the fluorophore is mixed with the laccase, its fluorescence shows a rapid decrease, which can be attributed to the catalytic effect of the enzyme on the oxidation of the fluorophore. However, this effect is delayed in the presence of a phenolic compound in a manner proportional to the concentration of the polyphenols. This biosensor allowed for catechol a LOD of 0.01 μM and a linear range of 0.08–5 μM.

**Figure 10:**
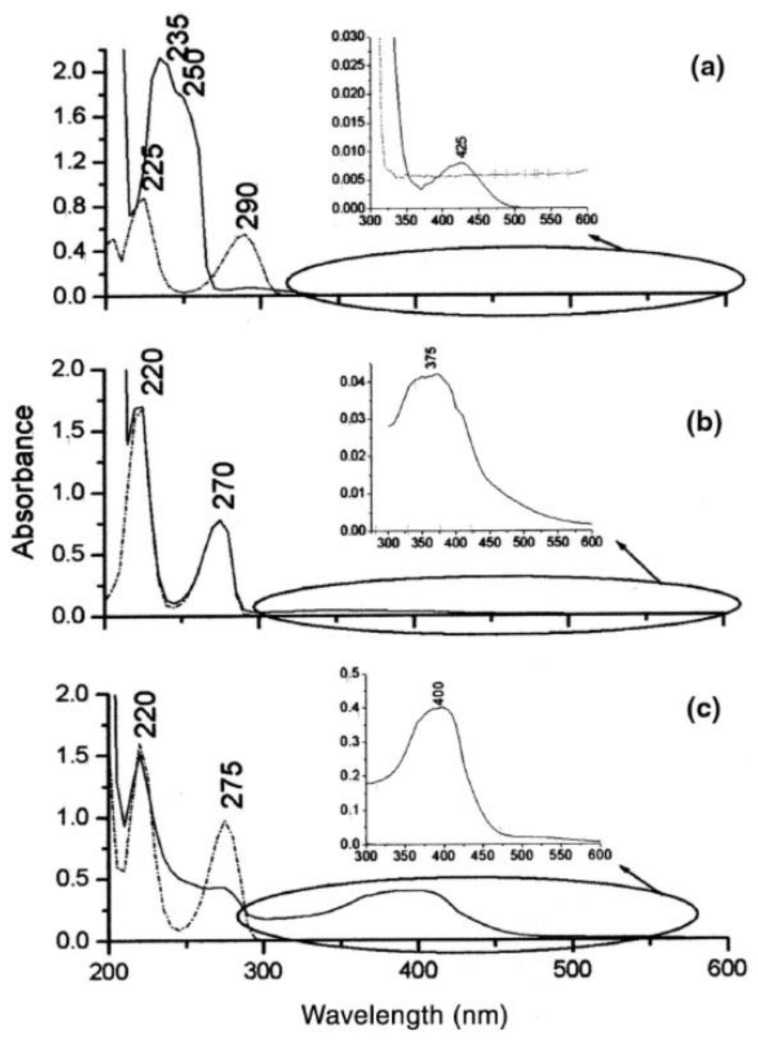
Experimental results obtained with an optical biosensor based on sol–gel immobilized laccase. (**a**) Absorption spectra of hydroquinone (dashed line) and laccase-reaction product (continuous line), (**b**) the same for resorcinol, (**c**) the same for catechol. In the different insets, the spectra in the 300–600 nm region. (Reprinted with permission from [59]).

Laccases and phenol reaction products present significant optical characteristics in UV and visible range that are routinely adopted for the development of spectrophotometric methods for measuring laccase activity [56]. These optical features can be suitably exploited for biosensing applications since laccase interacting with different phenols shows different optical absorption spectra. These differences can be useful for increasing the specificity of laccase-based biosensors [57]. To take advantage of the above-cited spectral properties, it is necessary to have optically transparent matrices for enzyme immobilization. Sol–gel technology can be a good choice for fabricating matrices for laccase immobilization with suitable chemical stability, optical transparency, and porosity [58,59]. The changes occurring in the optical absorption spectra of laccase reaction products at 425, 375, and 400 nm have been used to determine hydroquinone, resorcinol, and catechol concentrations, respectively (see Figure 10).

Owing to the slow response time of the hydroquinone–laccase reaction, the proposed optical biosensor was used for resorcinol and catechol. Linear ranges up to 1.4 and 0.2 mM and an LOD of 4.5 and 0.6 μM were evidenced for resorcinol and catechol, respectively. This type of biosensor is characterized by larger linear ranges, significant sensitivities, and good LODs when compared to other biosensors employing laccase from *Trametes versicolor*. Tap water samples spiked with a known amount of catechol and resorcinol were also employed for testing this biosensing device with real samples.

Another interesting optical biosensor for continuous monitoring of phenolic species in water was proposed by Jȩdrichowska et al. by using laccase from *Cerrena unicolor* immobilized by physical adsorption in low-temperature co-fired ceramics (LTTC) [60].

LTTC technology is a well-known technique largely employed in the industry. This kind of material has excellent physical and chemical properties, and three-dimensional structures can be easily obtained in the LTTC supports [61]. Microfluidic systems for sampling micro- and nanoliter volumes and optoelectronic components can be integrated in a single LTCC multilayer substrate [62]. In their paper, Jȩdrichowska et al. describe all the different steps required for biosensor fabrication and the optimization of the different parameters [60]. Scanning electron microscopy was used for visualizing the laccase, which was positioned onto the chemically modified substrate and the morphology of the deposited layer. Sensing measurements were performed in a flow-through system by evaluating the optical absorbance changes occurring in response to various concentrations of standard laccase assay substrate 2,20-azino-bis (3-ethylbenzothiazoline-6-sulfonic acid)-ABTS. In addition, the role of different sensor parameters, such as flow rate, optical source characteristics, and reproducibility, was investigated. According to the authors, the use of LTTC technology allows the realization of optical sensors characterized by significant advantages in terms of the sensitivity, precision, linearity, and simplicity of construction [60].

Very recently, Cano-Raya et al. proposed a new laccase-based optical biosensor for catechol concentration determination [63]. Laccase from *Trametes Versicolor* is attached to anionic polyamide 6 (PA6) porous microparticles placed in a Pebax MH1657 polymer binder that includes MBTH that can produce a colored product when it interacts with the o-benzoquinone produced by the enzymatic reaction of catechol. The analyte concentration is estimated by measuring the absorbance at 500 nm. The proposed biosensor is characterized by an LOD of 11 μM and a linear range up to 118 μM of catechol and has been challenged with spiked natural water samples from rivers and springs, showing a recovery rate varying in the 97–108% interval.

#### 3.1.2. Tyrosinase-Based Biosensors

Tyrosinase is a copper protein that catalyzes two successive reactions ([64] and references therein). In the first reaction, a hydroxyl group is added in the ortho position of a monophenolic compound, converting it into an o-diphenolic compound (monophenolase or cresolase activity). This diphenolic compound is subsequently oxidized into o-quinones by diphenolase or catecholase. Monophenols and diphenols are used by tyrosinase as a substrate. This enzyme can be found in different organisms, such as plants, animals, and fungi. The characteristics of this enzyme are different in size, sequence of amino acids, and glycosylation pattern [65]. Tyrosinase shows hydroxylase and catecholase activities due to histidine. In 2012 and 2017, two reviews prepared by Karim et al. [16] and Gui et al. [54] presented the optical biosensors reported in the literature.

In 1999, Russell and Burton [66] proposed a portable disposable biosensor using tyrosinase immobilized on a synthetic membrane for the detection and quantification of phenolic compounds in water. The enzyme produces changes of color in the solution, and these changes were found to be proportional to the phenolic substance concentration. The proposed biosensor is characterized by an LOD of 0.05 mg/L.

Using MBTH, Abdullah et al. developed also an optical biosensor based on the tyrosinase enzyme immobilized in a chitosan film similar to that previously described for the detection of phenol by laccase [67]. This biosensor exploits changes in the absorption spectra of the tyrosinase in the presence of MBTH and phenolic compounds. The authors investigated the response of this biosensor to different compounds and estimated the linear concentration range for 4-chlorophenol (2.5–50.0 μM), m-cresol (2.5–100.0 μM), and p-cresol (12.5–400.0 μM). The authors also reported interesting low LOD for the investigated phenolic substances.

Fiorentino et al. adopted a singular immobilization procedure in which ordered tyrosinase films deposited on an optical transparent support were immobilized by a “layer-by-layer” assembly, alternating the enzyme with the polycation polymer poly(dimethyldiallylammonium chloride) [68]. This procedure allowed a high loading of enzyme. The proposed biosensor was adopted for the detection of the o-diphenolic compound l-3,4-dihydroxyphenyl-alanine (l-DOPA) by means of absorption and fluorescence measurements. The developed device showed good repeatability and time stability. Using absorption measurements, an LOD equal to 23 μM and a linear response up to 350 μM was obtained; fluorescence measurements allow an LOD of 3 μM and a linear response in the range up to 10 μM.

Another example of biosensors exploiting a “layer-by-layer” immobilization was proposed by Alkasir et al. [69], who adopted this procedure for fabricating a colorimetric biosensor. The different layers are formed by chitosan and alginate polyelectrolytes deposited on a filter paper. The tyrosinase is embedded between these layers. This biosensor was used for the detection of phenol, BPA, catechol, and cresols [69]. The visual inspection of the color changes allows the detection of one of these substances. The color change is due to the specific binding of the quinone given by the enzymatic reaction to the multilayers of chitosan deposited on the paper. The digitalized approach was also used for more sensitive detection. The LOD was 0.86 ± 0.1 μg/L for each of the phenolic compounds studied. The proposed device showed very good time stability and was tested with real environmental samples.

**Figure 11:**
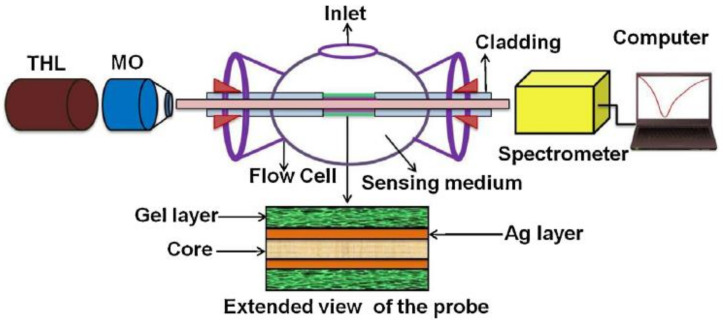
Schematic sketch of the experimental setup of an SPR-based fiber-optic phenol biosensor is shown. The fiber-optic probe is fixed in a small flow cell to enable the delivery and removal of aqueous samples of phenol around the sensing surface. Light from a tungsten–halogen lamp is coupled into the fiber. The spectrum of the transmitted power is recorded by using a spectrometer and a personal computer. (Reprinted with permission from [71]).

Microarray-based biosensor systems were proposed by Jang et al. for the determination of phenol using CdSe/ZnS quantum dots [70].Microarrays were based on poly(ethylene glycol)(PEG) hydrogel. They were prepared by photopatterninga solution containing PEG diacrylate (PEG-DA), a photoinitiator, and tyrosinase. Tyrosinase and QDs were entrapped within the hydrogel microarrays because of a photo-induced crosslinking. The obtained hydrogel microarray was characterized by a fluorescent signal whose intensity linearly decreases phenol concentration. The detection limit of this biosensor is 1.0 μM [70]. As we said before, the surface plasmon resonance (SPR) approach allows the realization of a highly performing sensing scheme. Singh et al. [71] presented an SPR-based fiber-optic biosensor for the detection of phenolic compounds in an aqueous solution [71], based on the use of the wavelength interrogation approach. Differently from the mentioned angular interrogation approach, when wavelength interrogation is used, the wavelength of light is varied, and the angle of incidence is kept constant and greater than the critical angle (wavelength interrogation). In this method, light from a polychromatic source is coupled into the input end of the fiber, and the spectrum of the transmitted power at the output end of the fiber is recorded. A dip at a specific wavelength is observed in the transmitted spectrum (this is the resonance wavelength): its position depends on the refractive index of the sensing medium around the metal layer, and, hence, the shift of the dip is related to the number of molecules captured by the substrate. In the reported case, a silver film was deposited on the core of an optical fiber and tyrosinase from lyophilized mushroom powder was immobilized using the gel entrapment technique. The experimental setup of this wavelength interrogation SPR-based fiber-optic phenol biosensor is shown in Figure 11.

The fiber-optic probe was attached to a small flow cell in which the aqueous solutions can be delivered to the sensing element and removed. The developed biosensor was used for determining the concentration of different phenolic compounds (phenol, catechol, m-cresol, and 4-chlorophenol).

**Figure 12:**
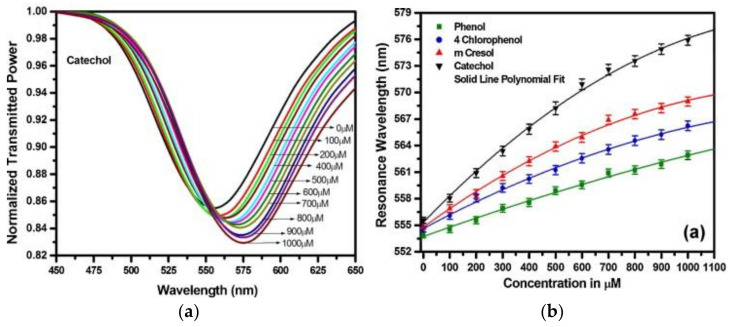
(**a**) Surface plasmon resonance spectra of a fiber-optic SPR probe for different concentrations of catechol, (**b**) Calibration curves obtained by measuring the variation in resonance wavelength for different concentrations of various phenol species. (Reprinted-adapted-with permission from [71]).

Aqueous samples of phenolic species with a variable concentration in the range of 0–1000 μM were examined. SPR spectra were collected for different concentrations, and the resonance wavelength showed a red shift when the concentration of the analyte increased. Representative calibration curves are reported in Figure 12.

The LODs were evaluated for catechol, m-cresol, 4-chlorophenol, and phenol and resulted to be around 11, 17, 25, and 38 μM, respectively. The authors evidenced that the characteristics (high sensitivity, wider operating range, reusability, and reproducibility of results) of the developed sensor make it suitable for practical applications. In these cases, it would be also possible to take advantage of miniaturization properties, low costs, online monitoring and remote sensing potentiality, immunity to electromagnetic fields, and biocompatibility.

An SPR approach has also been adopted very recently by Hashim et al. for realizing a biosensor for phenol solution in which tyrosinase is immobilized on graphene oxide thin film. The device is characterized by a sensitivity of 0.00193 μM^−1^ and a LOD of 1 μM with a linear range up to 100 μM [72,73].

As is evident from the few examples of enzymatic biosensors described in this section, the interest in this category of devices is always alive. New ideas will probably be developed by exploiting the properties of nano enzymes, a new class of nanomaterials that has peculiar physicochemical properties. Nanozymes can imitate natural enzymes and show similar properties. Their reactions are effective, fast, and highly selective. These characteristics make nano enzymes exceptionally good candidates for the development of new sensing and monitoring applications [74].

### 3.2. Immunosensors

Immunosensors are sensing devices composed of an antigen or antibody coupled to a transducer that can evidence the binding of complementary species. Antibodies are proteins that are produced by mammals in response to foreign elements (bacteria, viruses, chemicals, etc.). The analyte detection is very specific and can allow concentration measurements.

SPR technology can be advantageously coupled with antibody immobilization, and various examples of SPR-based immunosensors for the detection and monitoring of low-molecular-weight analytes for environmental applications are described by Shankaran et al. [75]. For SPR immunosensor fabrication, biomolecules, as antigen or antibody, are adsorbed on the gold surface, and all the changes occurring for these molecules or the different interaction occurring processes can be studied. The binding between the antibody and the analyte causes a change in the refractive index that induces a shift in the resonance angle that can be registered as previously described. These shifts allow the determination of bound analyte concentration and the evaluation of the affinity between analyte and antibody and give information about their association or dissociation processes. In Ref. [75], the advantages of SPR immunoassay are exhaustively described, and the different SPR immunoassay format characteristics are discussed.

Dostalek et al. [76] presented an example of an SPR-based immunosensor for different endocrine disruptors and, in particular, for 4-nonylphenol, which is widely used as detergents in both domestic and industrial products. The sensing scheme and the working principles are clearly described in Ref. [76], and for 4-nonylphenol, an LOD of 0.26 ng/mL is estimated. The obtained calibration curve allows the determination of concentration up to 4.4 ng/mL. Analytes can be detected in 45-min cycles, including 30-min incubation of antibodies with samples. The sensor is regenerable. The LOD is relevant in comparison with the maximum admissible concentrations in drinking water currently permitted by the regulatory authorities in the USA and EU.

**Figure 13:**
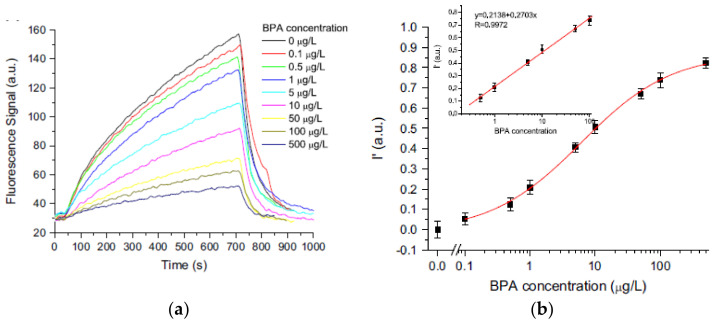
(**a**) Typical fluorescence curves signals obtained at various concentrations of BPA. (**b**) Logarithmic calibration curves for determination of BPA obtained by using an optofluidics-based immunosensor. In the inset, the linear relationship between BPA concentration and fluorescence intensity is shown. (Reprinted-adapted-with permission from [77]).

Long et al. [77] presented a highly sensitive and selective immunosensor for BPA detection that takes advantage of evanescent wave fiber-optic sensor and microfluidic technology for developing an all-fiber optofluidic-based bioassay platform [77]. In Figure 13, representative results are reported. The changes in fluorescence signal related to different BPA concentrations are used for realizing the calibration curve that shows a linear range between 0.5 μg/L and 1.0 μg/L and an LOD of 0.06 μg/L. This value is particularly appealing when compared with ELISA and amperometric biosensor performances. The biosensor was also tested with BPA-spiked samples, and the recovered data and the relative standard deviations were between 90–120% and 3.8–9.1%, respectively. The authors use the developed biosensing device also for investigating the BPA leaching of polycarbonate (PC) bottles of different brands. In fact, the residual and degraded BPA in this kind of bottle may migrate into food, especially at elevated temperatures for long periods [78]. The presented results show that the risk of BPA leaching from PC bottles is a real problem, and this approach can give a sensitive, rapid, on-site, real-time detection of BPA leaching.

### 3.3. Receptor-Based Biosensors

In 1998, Wright et al. [79] developed an SPR sensor with specific receptors for the detection of phenols in water. The authors synthesized some receptor molecules and immobilized them in gold or silver films. These films were deposited on glass slides mounted on a semicircular glass prism (refractive index 1.5151) and fastened to a small chamber connected to a peristaltic pump and waste reservoir. A p-polarized He: Ne laser was used for studying SPR response. The reported results evidenced the capability of this approach to detect and discriminate different phenolic species at low concentrations in aqueous media [79].

An example of this approach is represented by the device developed by Filik et al. for PAP employing CAL4 and reflectance measurements that we have previously described [25]. A typical result has been already reported in Figure 3. A linear calibration curve is obtained in the PAP concentration range of 0.5–5.5 ppm) with an LOD of 0.109 ppm. A response time of about 5 min is obtained for a stirred solution. The proposed sensor was also tested with several complex samples with spiked PAP, with recovered data ranging between 97 and 102%.

**Figure 14:**
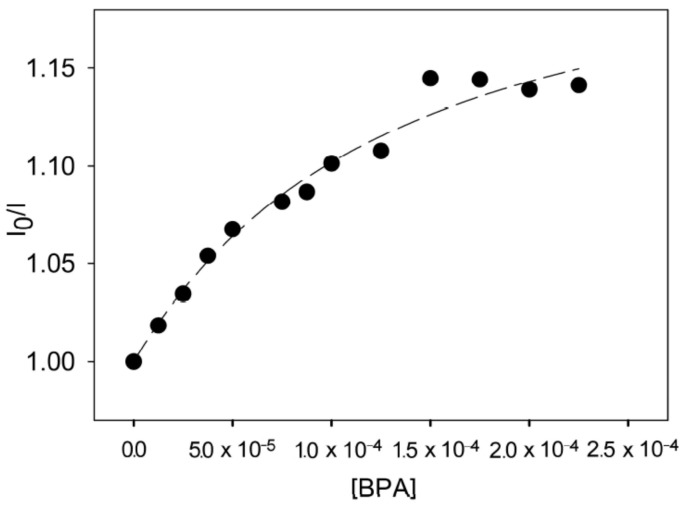
Calibration curve for different BPA concentration obtained by using the ratio of luminescence intensity of the [Ru (phen)2 L]2+ complex over luminescence intensity of the complex upon addition of increasing concentration. (Reprinted from [80] under Open Access conditions).

A very recent example of a receptor-based immunosensor has been developed by Conti et al. for BPA optical sensing by exploiting the luminescence emission of a new RuII complex that is able to bind BPA in an aqueous solution and to quench the luminescence emission of the core. The quenching effect is not remarkable, but the appropriately designed complexes can be used for determining BPA concentration in water. A linear calibration range up 50 μM BPA concentration has been obtained (see Figure 14) by using the luminescence quenching effect [80].

### 3.4. Nucleic Acids-Based Biosensors

Another very attractive class of biosensors is represented by the devices that exploit DNA as biosensing element. These biosensors can take advantage of the excellent stability of nucleic acids and the remarkable selectivity of the interaction of nucleic acids.

Yildirim et al. [81] proposed a portable, evanescent, wave fiber-optic DNA-based sensor for rapid, on-site detection of BPA with excellent sensitivity and selectivity. The authors covalently immobilized DNA on the optical fiber sensor surface. This biosensor uses an indirect competitive detection mode. For this sensing scheme, a pre-injection of BSA is used for avoiding the nonspecific binding to the sensor surface. After this step, the concentration of the remaining free aptamers becomes inversely proportional to that of BPA in the water sample. The sample solution is sent to the optical fiber sensor surface for allowing the generation of a useful fluorescence signal. The working parameters of the developed biosensors were investigated in detail. The authors reported a linear range for BPA from 2 nM to 100 nM with an LOD of 1.86 nM that becomes competitive with standard liquid chromatography detection results for BPA. Good reproducibility, stability, and selectivity for BPA detection were also demonstrated. The proposed sensor was successfully tested with wastewater samples [81].

Lim et al. [82] developed a palm-size NanoAptamer analyzer able to detect BPA at environmentally relevant concentrations (<1 ng/mL or ppb) with excellent sensing characteristics [82]. The presented biodevice uses a modified NanoGene assay [83] for BPA detection using magnetic beads for covalent bonding with a BPA-specific aptamer. After interaction with BPA, there is a decrease in the fluorescence signal that is proportional to the analyte concentration. The proposed biosensor showed a linear range for BPA from 0.0005 to 1 ng/mL. The BPA detection using this analyzer requires an incubation time of 30 min. This time can be positively compared with the time of other DNA aptamer methods for BPA detection that usually need incubation time lasting from 20 min to 8 h.

In 2019 Allsop et al. designed and fabricated another aptamer-based optical biosensor able to test BPA solutions in the concentration range from 10 nM to 1 fM. The presented device employs an array of gold nano-antennae that generate coupled localized surface plasmon (LSP) and are modified with an aptamer specifically for BPA detection [84]. The array of nano-antennae is assembled on a section of a standard telecommunication optical fiber. This configuration potentially enables multiplexing and remote sensing applications. Using a linear regression analysis, the authors can attain an extremely low LOD (330 ± 70 aM) that represents the lowest measured LOD.

### 3.5. Whole-Cells Biosensors

Optical microbial biosensors are devices that use microorganisms with an optical transducer to allow fast and accurate monitoring of the analytes of interest. Different researchers presented similar devices for applications in the field of environmental monitoring.

Mazhari and Agsar [23] proposed the use of *Streptomyces tuirus* DBZ39 synthesized on extracellular gold nanoparticles for the visual detection of phenol. The visual detection was improved by the addition of sodium sulphate, and the change of color occurred within 2 min. The proposed method was successfully tested with water samples from the effluents of fertilizer and distillery industries.

The same authors further exploited the properties of *Streptomyces tuirus* DBZ39 together with tyrosinase and gold nanoparticles for developing a paper biosensor for the detection of phenol from industrial water. The proposed biosensor can efficiently detect the changes in absorbance due to phenol presence thanks to the specific catalytic activity of the tyrosinase and the SPR contribution due to gold nanoparticles. This biosensor was tested with different types and quantities of phenolic constituents in various industrial effluents. The peculiar optical properties of gold nanoparticles increase the efficacy of tyrosinase for detecting phenol compounds [85].

### 3.6. Molecularly Imprinted Polymer-Based Sensors

Notwithstanding their synthetic origin, molecularly imprinted polymers (MIPs) are often considered biomimetic materials, and MIPs-based sensors are usually regarded as biosensors.

Griffete et al. exploit the characteristics of MIPs and photonic crystals for preparing a defect-embedded imprinted photonic polymer that is constituted by an ordered and interlinked three-dimensional microporous array [26]. In this structure, some nanocavities can interact with BPA using binding sites. Reflectance spectroscopy has been used to investigate the optical properties of the structure that are influenced by the interaction with BPA (see Figure 3 of Ref. [26]). The authors also demonstrate the selectivity and specificity of the developed MIP-based sensors for BPA solutions.

**Figure 15:**
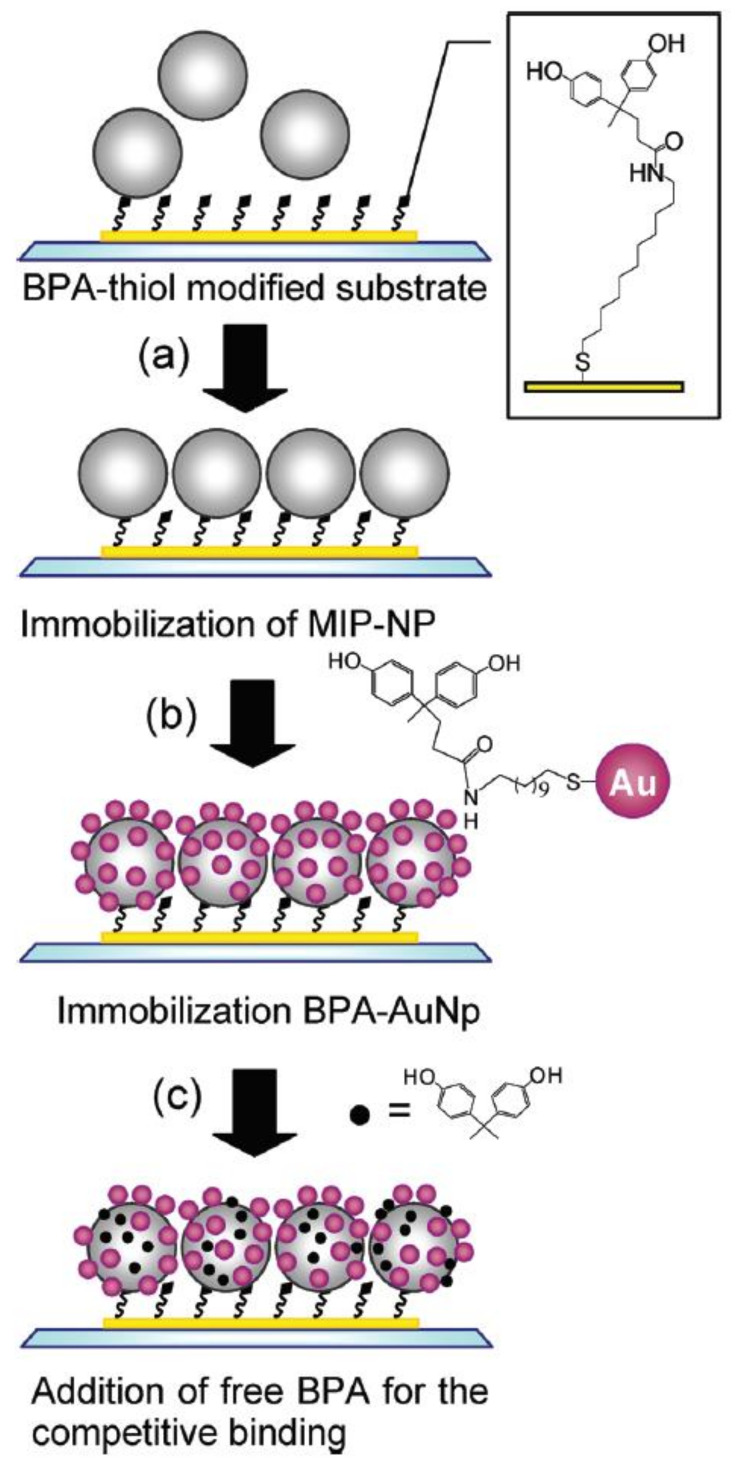
Schematic representation of the immobilization procedure of MIP-Np and BPA-Np on the sensor chip for the BPA detection. (Reprinted with permission from [86]. Copyright 2012 American Chemical Society).

Taguchi et al. developed a slab-type optical waveguide (s-OWG) and fabricated a microfluidic system [86]. On this OWG, consecutive parallel gold and silver bands are deposited. These can generate two individual SPR signals because of the difference in resonant reflection spectra of these metals. MIP nanoparticles were used as a recognition element for the BPA compound. In Figure 15, the immobilization procedure for preparing MIP nanoparticles and BPA grafted to gold nanoparticles on the sensor chip for the binding of free BPA is described. Peak shifts of SPR spectra by the addition of free BPA into MIP with immobilized nanoparticles were observed for BPA concentration varying in the range 0.1–2000 mM.

The detection of BPA is also the aim of other MIP-based sensors [87,88]. In Ref. [88], the use of black phosphorus and hollow-core anti-resonant fiber allowed two orders of magnitude enhancement of sensitivity in a fluorescence-sensing scheme. The simulated LOD was 1.69 pM, according to the calibration curve based on the IUPAC definition. The sensor was tested with real samples, such as water (collected from a lake near the campus of Beijing University of Technology) and human blood (see Figure 5 of Ref. [88]).

## 4. Sensors

In addition to the sensing schemes using biological transducers for detecting phenols that we described until now, optical techniques allow the implementation of chemical and/or physical sensors in which a chemical or physical property of a specific analyte is converted into a measurable optical signal that is proportional to the concentration of the analyte of interest. These sensors are less common for phenolic species detection due to their low sensitivity and selectivity, but currently, the development of new nanomaterials, such as gold, silver, and other metal nanoparticles; nanotubes; and quantum dots enables a significant improvement of these characteristics [89].

### 4.1. Optical Chemical Sensors

A clear classification of the different types of chemical optical sensors is shown in Figure 2 of Ref. [90]. The simplest sensing schemes are based on direct and reagent-mediated spectroscopic techniques. This framework includes methods based on the variations induced in the absorption or fluorescence signals of suitably designed inorganic probes [91,92,93]. Another relevant class of sensors is related to the design and fabrication of optical fiber chemical sensors that we have already mentioned in Section 2 [32,33]. A representative example of these devices has been developed by Wang et al. using a plastic optical fiber, a polymer membrane, a gold mirror, and a TiO_2_-based composite layer [38,94]. In particular, the relative variations of reflected light intensity are used as a working parameter. Phenol solutions at different concentrations were used for testing the sensor, and an LOD of 0.294·10^−3^ mg/L was obtained with a high selectivity.

### 4.2. Nanostructure-Based Sensors

As previously mentioned, the development of nanotechnology allowed the design and development of new sensing devices for phenolic species detection [95]. In 2008 Nezhad et al. presented an indirect colorimetric method for the optical detection of phenolic compounds exploiting the SPR band shown by gold nanoparticles [96]. By using the changes in the absorbance signal (typically occurring in a few tens of seconds) the authors were able to detect low concentrations of hydroquinone, catechol, and pyrogallol. Linear ranges from 7.0·10^−7^ to 1.0·10^−4^ M, 6.0·10^−6^ to 2.0·10^−4^ M, and 6.0·10^−7^ to 1.0·10^−4^ M were, respectively, obtained for the three above-mentioned phenolic species. The proposed method was also successfully tested with tap and river water samples.

Gold nanoparticles were also used for BPA detection by various researchers; for example, Ma et al. synthesized a diazonium carrying ligand monolayer film on the nanoparticles with the diazonium ions exposed on their surface [97]. The BPA-diazonium interaction causes a BPA concentration-dependent color change that can be employed for concentration determination. Also, for BPA, the gold nanoparticles-SPR method allows a fast response time (4 min), a broad linear range (0.1–4 nM), and a low LOD (0.02 nM). Table 1 of the cited paper also reports an interesting comparison among different methods available for BPA sensing.

In the last several years, semiconductor quantum dots, nanocubes, and nanorods have also attracted great interest in sensing applications due to their appealing physical and chemical properties [98,99]. Very representative examples of this class of devices are reported in Refs. [100,101,102,103,104]. In particular, Jaiswal et al. proposed a fast synthesis route of doped carbon nitride quantum dots for the detection of hydroquinone by photoluminescence quenching. They obtained an LOD of 50 nM and a linear range from 12 to 57.5 μM [102].

### 4.3. Photonic Crystal Fiber Sensors

Recently, Frazao et al. reviewed optical sensing applications of photonic crystal fibers. They revised the different approaches based on fiber Bragg gratings, long-period gratings, and interferometric structures. In addition, the role of nonlinear effects and the main sensing schemes for gaseous and liquid compounds were also discussed [105]. Using a photocatalytic long-period fiber grating (PLPFG), a fiber Bragg grating (FBG), a polymer membrane, an ultraviolet light, and microchannels, Zhong et al. developed a lab-on-a-chip device for phenol concentration sensing [38]. This approach is characterized by easy and fast in situ use, low consumption of agents and reagents, low costs, and high sensitivity. The PLPFG component is a three-layer structure in which the refractive index of the cladding layer is less than that of the core, and both are less than the refractive index of the coating layer. This is a photocatalytic film for UV-visible-driven photocatalytic degradation of phenol. The LPFG enhances the evanescent wave absorption and shifts the central wavelength due to the interaction between the evanescent wave and the analyte. The developed sensor is characterized by a linear performance in a large range of phenol concentrations (7.5 μg/L to 100 mg/L). It can operate at pH values and temperatures ranging from 2.0 to 14.0 and from 10 °C to 48 °C, respectively.

A sensor for BPA and bisphenol S (BPS) based on photonic crystal technology was proposed by Niger et al. employing a dodecagonal photonic crystal fiber structure having a floral pattern in the first cladding layer [106]. Using this approach, the authors reported a relative sensitivity of 97.6% and 94.9%, respectively, for BPA and BPS.

### 4.4. Sensing Schemes Using Vibrational Spectroscopies

As described in Section 2, vibrational spectroscopies have been largely employed for designing and developing a huge number of devices for sensing different phenolic compounds. In 1984, the pioneering work of Marley et al. was devoted to evaluating the possibility to use RS for the quantitative analysis of six compounds (phenol, o-chlorophenol, 2,4-dichlorophenol, 2,4,6-trichlorophenol, 2-chloro-5-methylphenol and 2-chloro-4-nitrophenol) in water [107]. The authors used two different methods for the quantitative determination of concentrations, namely peak area measurement and cross-correlation. Both methods were applied to data after Savitzky-Golay smoothing and after correction for internal standard fluctuations. Areas were measured on selected bands in the spectra, and cross-correlation operation was accomplished on a complete set of spectra for a given compound, eliminating frequency components from both the high and low ends of the set. LODs ranged from 0.3 ppm to 100 ppm, depending on the compound.

**Figure 16:**
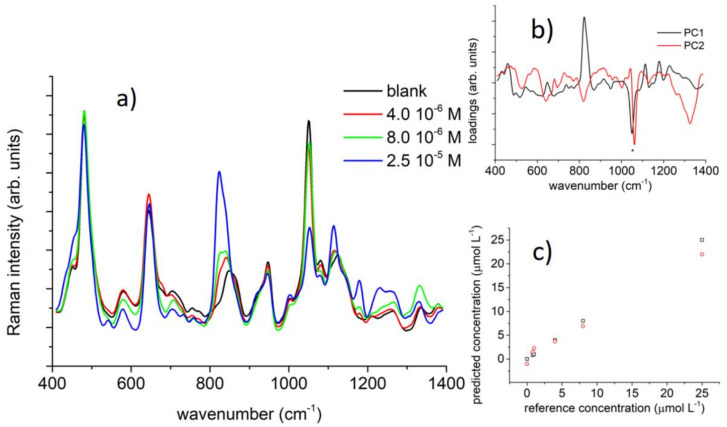
(**a**) BPA SERS spectra as a function of the BPA molar concentration. (**b**) Loadings for the first two components in the PCA analysis on the BPA SERS data. (**c**) Calibration curve as obtained by the partial least square regression analysis on SERS data. (Reprinted from [112] under Open Access conditions).

The use of properly designed nanostructured substrates has allowed the implementation of a certain number of sensing devices for BPA [108,109,110,111,112,113,114,115,116,117,118] by means of SERS. Typically, these devices exploit silver or gold nanoparticles that can be functionalized with organic groups to enhance Raman signal intensity and, consequently, the sensitivity for BPA or other phenolic substances concentration determination. In particular, Roschi et al. employed silver nanoparticles functionalized with thiolated-cyclodextrin (CD-SH) for the detection of bisphenols (BPs) A, B, and S. Using multivariate analysis of the SERS data, the LOD for BPs was estimated at about 10^−7^ M, in the range of the tens of ppb (see Figure 16) [112].

## 5. Conclusions

The danger of phenolic compounds and their tendency to remain present in the environment motivates the intense search for new methods for their detection and measurement of their concentrations. In this paper, we intended to highlight the relevant role that optical techniques can play in this framework. For this reason, we focused our attention on UV-vis fluorescence, reflectance, and absorption experimental approaches, on the different uses of optical fibers and Bragg gratings and on SPR and vibrational spectroscopies methods. In addition, we revised some representative applications of these techniques in developing new sensors for some phenolic compounds especially present in water.

In Table S2, we summarized the most relevant working parameter of these optical sensors. In particular, their sensitivity, linear range, LOD, and response time have been reported. From the inspection of this Table, it is evident that the largest number of optical sensing schemes among those discussed in this review has been proposed for bisphenol A and phenol. More conventional optical techniques, such as absorption and UV-vis fluorescence, are still adopted, often using innovative biocomponents. However, it is also evident that more novel optical techniques, such as SPR, SERS, and SERRS (Surface-Enhanced-Resonant Raman Spectroscopy), with the help of sophisticated nanostructures, are making their way. Despite the difficulty of a rigorous comparison due to the different experimental conditions, it can be seen that the use of advanced new biocomponents and new optical techniques is offering ever more performing working parameters.

Although limited to the most common techniques and not particularly complete, this review certainly confirms the important role played by optical techniques for the development of sensitive, fast, and easy-to-use biosensors and physical and chemical sensing schemes for phenolic species monitoring.

## Data Availability

Not applicable.

## Supplementary Materials

The following are available online at https://www.mdpi.com/article/10.3390/s21227563/s1, Table S1: Permissible concentration limits for different phenolic compounds, Table S2: Relevant working parameters for the optical sensors for the determination of phenolic compounds in environmental applications discussed in the present paper.
