# Supplementary material for: Advanced Optical Sensing of Phenolic Compounds for Environmental Applications
Source: Sensors (Basel). 2021 Nov 14;21(22):7563. doi: 10.3390/s21227563 (PMC8619556; doi:10.3390/s21227563)
Supplement: Supplementary file 1 [file sensors-21-07563-s001.zip › sensors-1439329-supplementary.pdf]

SUPPLEMENTARY MATERIAL

Phenolic compound	Permissible concentration limit	
Phenol	1 μg/L	0.010 μM
Nonylphenol	2.0 μg/L	0.009 μM
Pentachlorophenol	1 μg/L	0.004 μM
Total cresol	200 μg/L	1.85 μM
BPA	4 μg/kg bw/day (Tolerable daily intake)	

Table S1. Permissible concentration limits for different phenolic compounds. Data are taken from EPA website https://www.epa.gov.

Table S2 Relevant working parameters for the optical sensors for the determination of phenolic compounds in environmental applications discussed in the present paper.

**Phenolic compound**	**Optical technique**	**Sensivity**	**Linear range**	**LOD**	**Response time**	**Ref.**
Bisphenol A	Absorption spectroscopy	0.1/ppm	0.23-5 ppm	0.23 ppm	10 min	24
	Reflectance spectroscopy					26
	Color intensity		Up to 25 μg/L	0.86 (±0.1) μg/L	6-17 min	69
	Fluorescence spectroscopy		0.5–100 μg/L	0.06 μg/L		77
	Fluorescence spectroscopy		2 nM -100 nM	1.86 nM 0.45 ng mL^{-1}	~400 s	81
	Fluorescence spectroscopy		0.0005-1.0 ng/mL (ppb)			82
	SPR		1 fM -1 nM	330 ± 70 aM		84
	SPR		0.1-2000 mM			86
	Fluorescence spectroscopy		3×10^{-9}–5×10^{-6} g mL^{-1}	1.7×10^{-9} g mL^{-1}	~3 min	87
	SPR		0.1–4 nM	0.02 nM	4 min	97
	Fluorescence spectroscopy		10 - 600 pM.	1.69 pM	~10 min	88
	SERS			3 nM		108
	SERS	50 (μg $L^{-1})^{-1}$	2 - 100 μg L^{-1}	1 μg L^{-1}	30 min	110
	SERS		0.01–1 ng mL^{-1}	2.8 pg mL^{-1}	15 min	111
	SERS		Up to 10 μM L^{-1}	~10^{-7} M		112
	SERRS			~0.1 ppb (0.1 μg/kg)		114

Catechol	Absorption spectroscopy		0.5–8.0 mM	0.33 mM	10 min.	53
	Time course absorption spectroscopy	0.521 ± 0.016 Min^{-1} mM^{-1}	Up to 0.2 mM	0.6 µM	3 min	59
	Absorption spectroscopy		Up to118 µM	11 µM	30 min	63
	Color intensity		Up to 25 µg/L	0.86 (±0.1) µg/L	6-17 min	69
	SPR	0.032 nm μM^{-1}		11 µM		71
	SPR		6.0×10^{-6} - 2.0×10^{-4}M	2.5×10^{-6} M		96
Phenol	Absorption spectroscopy		0.02-012 mM		4-5 min	23
	Transmission spectroscopy	0.195×10^{-3}(mg /L)$^{-1}$		40 µg L^{-1}		37
	Fiber Bragg grating	−0.0492 nm (mgL$^{-1)-1}$	7.5 µg L^{-1}-100 mg L^{-1}	7.5 µg L^{-1} (79.7 nM)	~395 s	38
	Absorption spectroscopy		9.79- 750 mM	0.109 mM		54
	Colorimetric assay	1.78	Up to 100 mgL^{-1}		3 h	66
	Absorption spectroscopy	1.78 Abs mM^{-1}	2.5–70.0 µM	1 µM	15 min	67
	Color intensity		Up to 25 µg/L	0.86 (±0.1) µg/L	6-17 min	69
	SPR	0.012 nm μM^{-1}		38 µM		71
	SPR	0.00234° μM^{-1}	0-20 µM	1 µM		72
	SPR	0.00193° μM^{-1}	Up to 100 µM	1 µM		73
	Reflection spectroscopy	0.294×10^{-3} (mg · $L^{-1})^{-1}$	10-100 mg L^{-1}	30 µg L^{-1}	~70 min	94

	NIR fluorescence spectroscopy		Up to 25 µM L^{-1}	0.05 µM L^{-1}		100
	Raman spectroscopy		50-500 mg L^{-1}	25 mg L^{-1}		107
	SERS			1.9×10^{-10} M		113
	SERS			10^{-5}–10^{-6} M.		118
p-aminophenol	Reflectance spectroscopy		0.5–5.5 ppm	0.109 ppm	5 min	25
Resorcinol	Time-course absorption spectroscopy	0.075 ± 0.001 Min^{-1} mM^{-1}	Up to 1.4 mM	4.5 µM	3 min	59
m-cresol	Absorption spectroscopy	1.73 Abs mM^{-1}	2.5–100.0 µM	1 µM	15 min	67
	Color intensity		Up to 25 µg/L	0.86 (±0.1) µg/L	6-17 min	69
	SPR	0.022 nm μM^{-1}		17 µM		71
p-cresol	Colorimetric assay		Up to $100 mgL^{-1}$		3h	66
	Absorption spectroscopy	0.31 Abs mM^{-1}	12.5–400.0 µM	3 µM	15 min	67
	Color intensity		Up to 25 µg/L	0.86 (±0.1) µg/L	6-17 min	69
4-chlorophenol	Absorption spectroscopy	2.04 Abs mM^{-1}	2.5–50.0 µM	0.9 µM	15 min	67
	SPR	0.016 nm μM^{-1}		25 µM		71
o-diphenol	Absorption spectroscopy	0.184 Abs $min^{-1} mM^{-1}$	Up to 350 µM	23 µM	2 min	68
	Fluorescence spectroscopy		Up to tens of µM	3 µM	2 min	68

Hydroquinone	SPR		7.0×10^{-7}-1.0×10^{-4} M	5.3×10^{-7} M		96
	Photo luminescence	0.021 μM^{-1}	12 - 57.5 μM	50 nM		102
Pyrogallol	SPR		6.0×10^{-7} - 1.0×10^{-4} M	3.2×10^{-7} M		96
p-nitrophenol	Photo luminescence	0.04918 μM^{-1}	3 - 96 μM	4.09 μM		103
Thiophenol	NIR fluorescence spectroscopy	0.0939 μM^{-1}	0–10 μM	0.22 μM	3 min	93
